# A Model Court and Trial

**Published:** 1910-11

**Authors:** 


### A Model Court and Trial

THE trial of Hawley H. Crippen, for the murder of his wife, Belle Elmore, held in London, October 18 to 22, 1910, was one of the most remarkable in recent criminal jurisprudence, and may well serve as a model for the guidance of American Courts in murder trials. Several points become impressive when we remember the "law's delay" in similar trials in this country.

Lord Chief Justice Alverstone chose to preside owing to the importance of the case. He did not ask anybody, but assumed the responsibility himself. The jury was obtained expeditiously and the time was not wasted by irrelevant discussion. The Lord Chief Justice assisted the jury by incisive questions, especially when expert witnesses were likely to bewilder them with technicalities. He also protected witnesses exposed to cross examination, and was helpful to Crippen himself at critical stages of the testimony.

Counsel on both sides were kept under restraint, and were not allowed to abuse their privileges by introducing unfair comment when they were examining witnesses or dealing with the experts. The manners of counsel were perfect and the Lord Chief Justice, both in the general conduct of the case and in his lucid charge to the jury, was the embodiment of impartiality.

The accused was defended with ability and the prisoner himself sought to reinforce his eloquent counsel by a prolonged exhibition of coolness and nerve, and even when the verdict was rendered protested his innocence with blanched face. The crown solicitors have done their work with amazing thoroughness and the chain of circumstantial evidence was irrefragable.

The jury was out just thirty minutes, when it returned and announced that it had found the defendant guilty. Lord Chief Justice Alverstone asked the prisoner if he had anything to say. Crippen replied in a low voice: "I still protest my innocence."

The chief justice then donned the black cap that had rested near him throughout the trial and pronounced the sentence of death. Addressing the condemned man, Lord Alverstone said:

"You have been convicted on evidence which can leave no doubt in the mind of any reasonable man that you cruelly murdered your wife and then mutilated her body.

"I advise you to entertain no hope that you will escape the consequences of your crime. I implore you to make your peace with Almighty God."

The trial of this celebrated case, in which the public has taken the keenest possible interest, has shown that the criminal procedure in the English courts is wellnigh perfect in dignity and efficiency, and that the rights of the individual, even when he is a monster and outcast, are protected with absolute impartiality.

We have invited particular attention to this case for the purpose of accentuating the methods of the Lord Chief Justice in dealing with the expert witnesses; when they were befogging the jury, he checked them, and when the lawyers were misleading the jury through these questions to the experts they received rebuke.

---

A MOST surprising report of epidemic infantile paralysis, poliomyelitis, was recently sent out from Harrisburg, Pa. It states that, according to reports received by the State Department of Health, there were 658 cases of infantile paralysis in forty-five of the sixty-seven counties of Pennsylvania. The largest number was in Lancaster County, where there were 135 cases. Philadelphia reported seventy-nine cases. Infantile paralysis was recently made a reportable disease in the state of Pennsylvania.

---

DR. ALEXUS CARRELL, according to a despatch from Baltimore, dated October 18, 1910, has made an important discovery relating to the growth of healthy tissues of the human system. The work is being done at the Rockefeller Institute for Medical Research at Baltimore.

Dr. Carrell's discovery involves a method for removal from the human body portions of the stomach, bloodvessels, skin, bone and practically every other tissue and making them grow much as they did in the human form to which they belonged.

The Rockefeller institution's attention has been directed for some time to the science of germ cultivation, the development of which within the last few years has been a signal triumph to medicine. Dr. Carrell, who had paid much attention to the cultivation of the germs of disease some time ago, began to apply himself especially to the growth of healthy tissues of the human system after they had been removed from the body.

He tried his experiments with tissues from the stomach, with tissues from bloodvessels, cartilage and bone, and in the end his efforts were rewarded by success. He has kept portions of the body alive as long as three weeks after they were taken from the person to whom they belonged. Other experiments have resulted in the tissues being kept alive for from a week to two weeks. The methods of cultivating the living body or portions of it is somewhat similar to the cultivation of disease germs.

REPORTS from Russia, Italy and Germany to the Public Health and Marine Hospital Service indicate that the epidemic of cholera is abating. Officers of the foreign corps report they have no doubt the present epidemic originated in Odessa and that rats were the cause. Nearly every case they have discovered in the Russian city was that of a person who lived or worked on the ground floor of a building infested by rats.

From Russia the officers trailed the plague into Italy. A party of Russian gypsies fleeing from the police carried it there and started the epidemic when they used the vessels at a public well for washing clothing. The infection quickly spread.

Every quarantine official in the United States has been on the alert to prevent a case of cholera from being brought into this country. Dr. Doty, quarantine officer at New York, has held up three ships from Naples and other Italian ports. The passengers complain, of course, because of the delay occasioned, but it is only through such energy and efficiency that infectious diseases of extreme virulence like cholera are headed off.

